# Precision Onion Skinning Technique for Transjugular Intrahepatic Portosystemic Shunt Revision

**DOI:** 10.7759/cureus.3180

**Published:** 2018-08-22

**Authors:** Jerry Matteo, Damian Caci, Erik Soule, James Cunningham, Preston Hood

**Affiliations:** 1 Interventional Radiology, University of Florida College of Medicine, Jacksonville, USA; 2 Interventional Radiology, University of Florida Health, Jacksonville, USA

**Keywords:** tips, reduction, revision, post, precision, onion skin, portosystemic shunt, intrahepatic portosystemic shunt

## Abstract

A transjugular intrahepatic portosystemic shunt is the standard of care for complications of portal hypertension, such as variceal bleeding, refractory ascites, and hepatic hydrothorax. Hepatic encephalopathy, hepatic insufficiency, and right heart failure are the major complications after shunt creation. If medical management is unsuccessful, the interventionalist is consulted to close/revise the shunt. Closure of the shunt results in a dangerously abrupt increase in portal pressure, increasing risk for life-threatening variceal bleeding. Methods for revising these shunts are reported, which result in coarse adjustments in shunt diameter, causing rapid changes in portosystemic gradients. Our method for shunt revision utilizes carefully sized covered stents deployed in a controlled “onion skin” fashion to produce a narrowing within the hepatic venous limb of the shunt to precisely calibrate the desired portosystemic gradient.

## Introduction

In this article, we report a novel technique utilizing commercially available devices to precisely and incrementally adjust the portosystemic gradient across a transjugular intrahepatic portosystemic shunt (TIPS) in patients with cardiac overload or hepatic encephalopathy. Hepatic encephalopathy, hepatic insufficiency, and high-output right heart failure are among the major complications after successful TIPS creation [1]. We illustrate a novel technique for transjugular intrahepatic portosystemic shunt revision. Precision onion skin technique (POST) allows for a precise stepwise alteration of the hemodynamics across a TIPS to achieve a target portosystemic gradient in a patient with shunt-related complications, such as congestive overload or refractory hepatic encephalopathy.

## Case presentation

We illustrate this method by presenting a 54-year-old male with alcoholic and hepatitis C cirrhosis complicated by portal hypertension, refractory ascites, and hepatic hydrothorax. His recent medical history was significant for ST-elevation myocardial infarction treated by percutaneous angioplasty, pericarditis, hemopericardium treated by the pericardial window, and hepatic encephalopathy. With medical management, the patient’s ammonia level was successfully decreased and his encephalopathy had resolved, however, his ascites and hydrothorax were refractory to aggressive medical management. Interventional radiology was consulted by cardiothoracic surgery to be evaluated for TIPS creation for definitively treating his refractory ascites and hepatic hydrothorax. At the time of evaluation, there was no encephalopathy (ammonia 40 ug/dl), his Model for End-Stage Liver Disease (MELD) score was 12, and his left ventricular ejection fraction was greater than 75% by echocardiography. Therefore, he was considered an acceptable risk for TIPS creation.

A TIPS was successfully created from a right hepatic vein to a right portal vein with a 10 centimeter (cm) x 6/2 cm Viatorr stent graft (WL Gore and Associates, Flagstaff, AZ, USA). This reduced the portosystemic gradient from 11 mmHg to the desired 5 mmHg. Following this, the patient’s condition improved and he was discharged home in stable condition.

One month later, the patient presented to the emergency department with decompensated congestive heart failure (CHF). The patient was admitted to the medical intensive care unit (MICU) and was aggressively diuresed. A right heart catheterization by the MICU team revealed elevated pressures (pulmonary artery mean 36 mmHg, pulmonary wedge pressure 28 mmHg) suggesting high output right heart failure likely aggravated by the TIPS. Therefore, the patient was referred to vascular and interventional radiology for TIPS revision.

**Figure 1:**
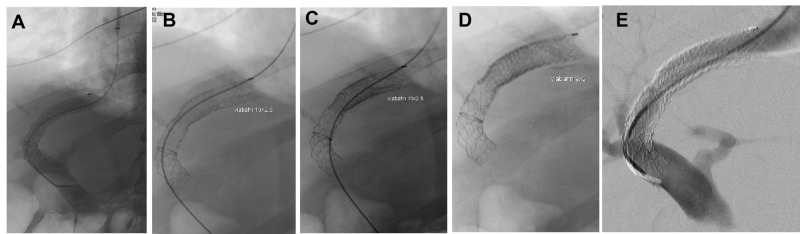
TIPS revision angiogram of a 54-year-old male with heart failure following TIPS placement. A) Pre-revision shunt angiogram demonstrates a hepatopedal portal venous flow with a widely patent shunt. The portosystemic gradient measured 5 mmHg. B) The first revision layer, a Viabahn stent graft 10 mm x 2.5 cm was deployed within the hepatic venous outflow limb of the Viatorr shunt. The portosystemic gradient increased to 6 mmHg. C) The second revision layer, a Viabahn stent graft 10 mm x 2.5 cm was deployed and positioned flush with the first layer, increasing the portosystemic gradient to 7 mmHg. D) The third revision layer, a Viabahn stent graft 9 mm x 5 cm was deployed and positioned with proximal and distal overlap to create an hourglass shape, increasing the portosystemic gradient to 9 mmHg. E) With the portosystemic gradient goal reached, the final shunt angiogram demonstrates a smooth hourglass shunt narrowing. TIPS: transjugular intrahepatic portosystemic shunt; Viabahn: WL Gore and Associates, Flagstaff, AZ, USA;Viatorr: WL Gore and Associates, Flagstaff, AZ, USA

TIPS revision was performed using a right internal jugular vein (IJV) approach. The percutaneous access of the right IJV was performed using a micropuncture kit and upgraded to an 11 French 10 cm sheath (Terumo, Somerset, NJ, USA). Portography was performed with a 5 French pigtail catheter, which showed a widely patent portal vein with hepatopedal flow through the existing TIPS (Figure 1).

**Figure 2:**
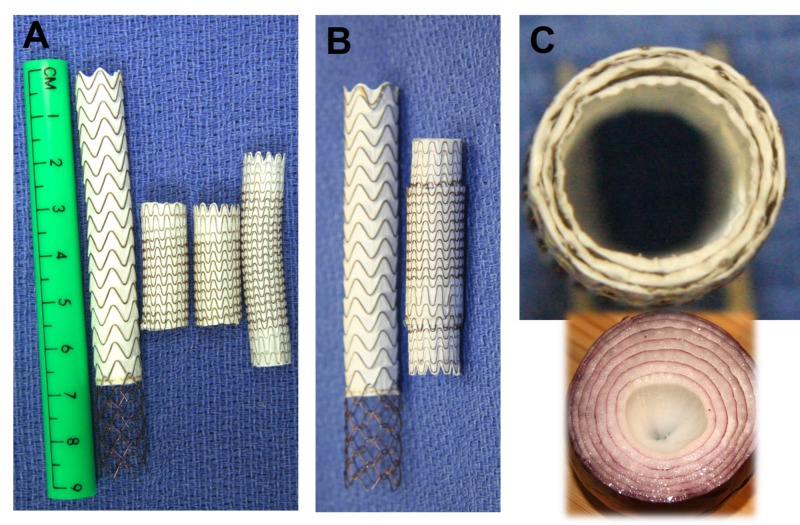
A) Shows a back table view of all the stents used in the procedure from left to right: 10 mm x 6 cm covered/2 cm uncovered Viatorr TIPS stent, two 10 mm x 2.5 cm Viabahn stents and a 9 mm x 5 cm Viabahn stent graft. B) Shows the Viatorr on the left with the 9 mm x 5 cm stent inside the two 10 mm x 2.5 cm stents on the right. C) Shows the cross-sectional view of the four separate stents forming the “onion skin” configuration used to create the smooth narrowing. TIPS: transjugular intrahepatic portosystemic shunt; Viabahn: WL Gore and Associates, Flagstaff, AZ, USA; Viatorr: WL Gore and Associates, Flagstaff, AZ, USA

Following this, pressures within the portal vein and right atrium measured 25 mmHg and 20 mmHg, respectively, for a portosystemic gradient of 5 mmHg. At this point, the decision was made to revise the TIPS with precision using a piecemeal placement of the stent grafts, thus restricting the overloaded cardiac flow and increasing the portosystemic gradient while minimizing the risk of causing variceal hemorrhage. The goal was to precisely bring the portosystemic gradient to 9 mmHg. At the time of revision, his ammonia was 63 and MELD score was 14. Three Viabahn (Viabahn; W L Gore and Associates, Flagstaff, AZ, USA) stents (two 10 mm x 2.5 cm stents followed by one 9 mm x 5 cm stent) were deployed in a sequential “onion skin” fashion to create a customized hourglass stenosis within the hepatic venous segment of the TIPS. Back table images are shown, demonstrating the relative sizes and configuration of the stent grafts (Figure 2).

**Figure 3:**
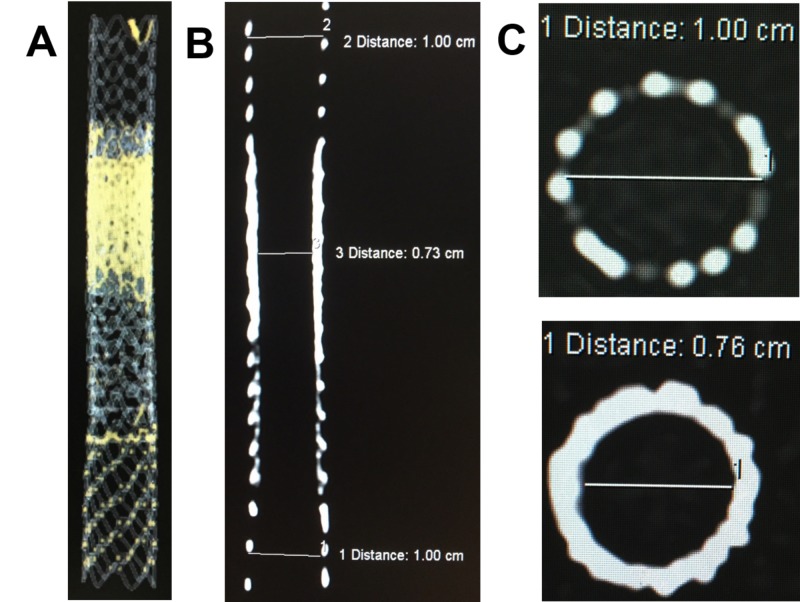
A) 3D CT image of the Viatorr and Viabahn stents laminated. B) Coronal CT image showing the smooth hourglass tapering from the onion skin technique. C) Shows the axial CT image of a 10 mm Viatorr stent (top) and axial CT image through onion skin layers of Viabahn stent grafts with a narrowing of the lumen to 0.76 mm (bottom). CT: computed tomography; Viabahn: WL Gore and Associates, Flagstaff, AZ, USA; Viatorr: WL Gore and Associates, Flagstaff, AZ, USA

Computer tomography (CT) acquisitions of the stent grafts were captured with three-dimension and multiplanar reformatting for a further illustration of the stent grafts in an “onion skin” configuration (Figure 3).

Portosystemic gradients after each stent layer measured 6 mmHg, 7 mmHg, and 9 mmHg, respectively. The right atrial pressure was decreased from 25 mmHg to 10 mmHg.

**Figure 4:**
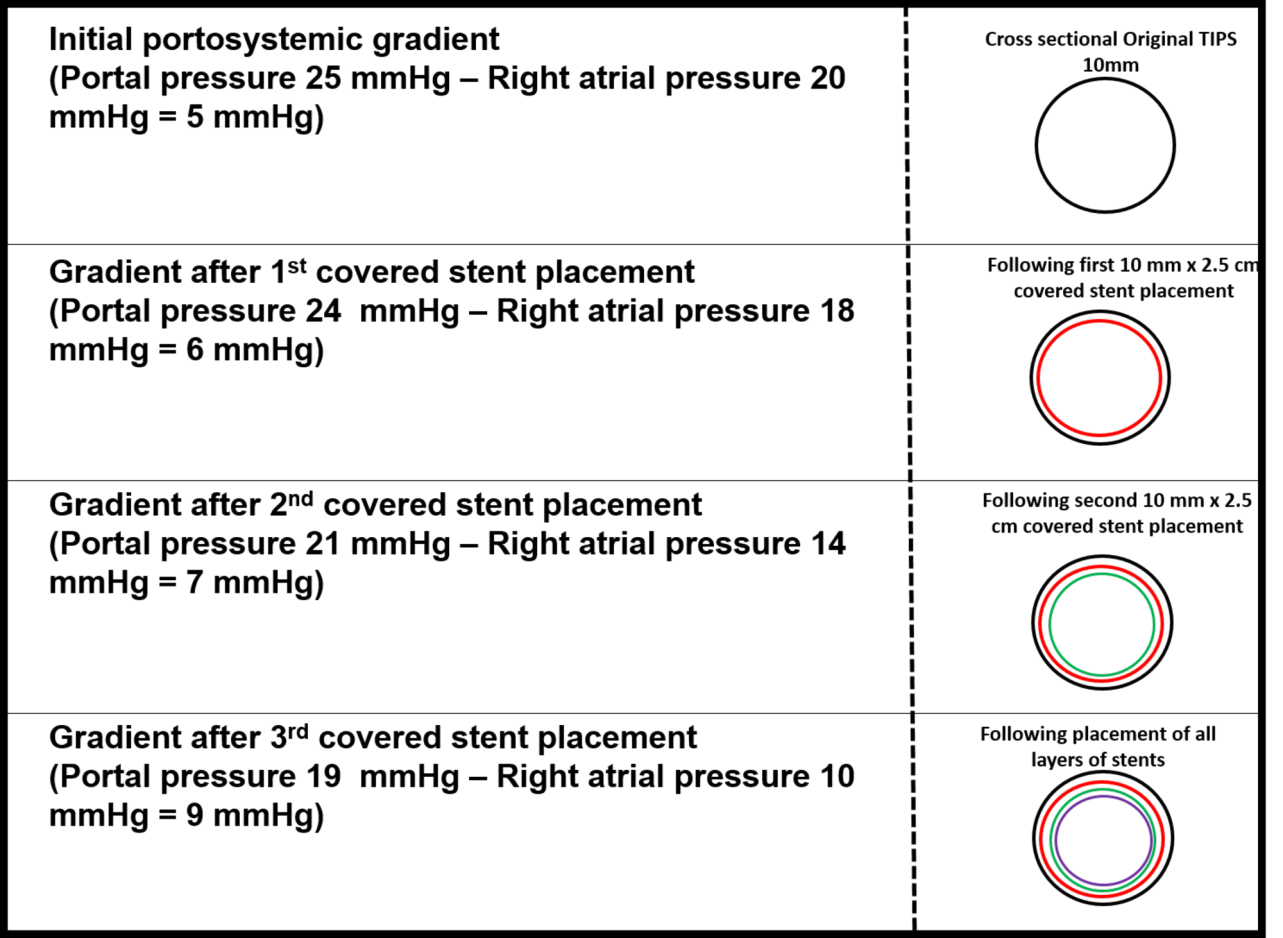
Schematic chart showing the correlation between the portal and right atrial pressures after each layer of stent graft material applied to create the narrowing in a 54-year-old male during TIPS revision using the precision onion skinning technique. TIPS: transjugular intrahepatic portosystemic shunt

A chart was constructed correlating the portal and right atrial pressures after each layer of stent graft material used to create the narrowing of the lumen (Figure 4).

The patient was discharged home in a stable condition without clinical evidence of CHF.

## Discussion

**Figure 5:**
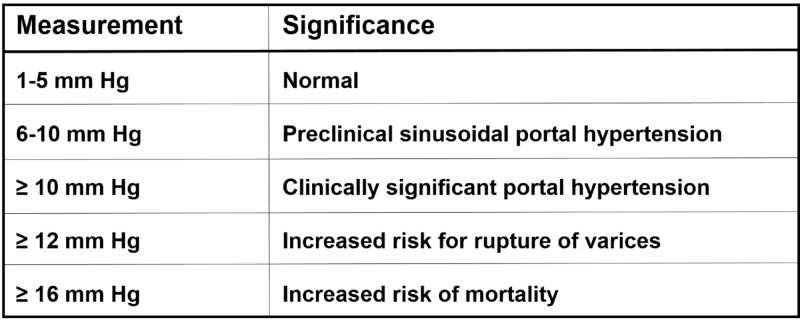
Chart shows the correlation of portosystemic pressure gradients with clinically significant portal hypertension.

The hemodynamic changes after TIPS creation result in the preferential flow of portal blood through the TIPS, which can elevate pulmonary artery pressure, right atrial pressure, cardiac index, and pulmonary vascular resistance. Although the patient presented in this article had a satisfactory pre-treatment echocardiogram, the hemodynamic changes after TIPS led to an acute decompensated high-output right heart failure. Medical management with aggressive diuresis, high-pressure O_2_ via non-rebreather mask, and light sedation with morphine was unsuccessful. Therefore, interventional radiology was consulted to close or revise the TIPS. Patients with CHF following TIPS creation usually present clinically with shortness of breath. Chest radiographs will reveal findings compatible with CHF such as enlargement of the cardiac silhouette and the central engorgement of the central pulmonary vasculature. One of the most important aspects of endovascular TIPS revision using stent grafts is achieving a desired portosystemic pressure. Mild portosystemic hypertension is 6-10 mmHg while clinically significant portal hypertension is 11-15 mmHg (Figure 5) [2].

The case presented describes a portosystemic pressure gradient of 5 mmHg, resulting in post-procedure decompensated CHF. Therefore, the portosystemic gradient of 9 mmHg was chosen as the target to decrease the cardiac overload while avoiding clinically significant portal hypertension.

Several elegant techniques for reducing TIPS have been reported. The current options for TIPS revision in patients with cardiac overload include complete closure of the shunt, placing inline, pre-constrained stents within the shunt as well as parallel partially constrained stents within the shunt [3-4]. However, these options do not allow for precisely controlled portosystemic gradients and, therefore, can cause abrupt hemodynamic changes. In a patient at risk for variceal hemorrhage, this can be a devastating outcome [5]. Many of the reported methods for TIPS revision, such as suture-constrained and parallel-constrained stent placements, can result in coarse adjustments in shunt diameter, resulting in rapid changes in a portosystemic pressure gradient [6]. Moreover, the closure of the TIPS by coil or vascular plug embolization could result in a dangerously abrupt increase in portal venous pressure [7], putting the patient at risk for life-threatening variceal bleeding.

Additionally, the described techniques in the literature require highly trained skills and modified materials, thus making them somewhat esoteric and making the results nearly impossible to reproduce with dependable accuracy. Therefore, a TIPS revision method was necessary to gradually, incrementally, and precisely reduce the portosystemic shunt. In contrast, the technique we report is relatively simple, utilizes off-the-shelf materials, and offers reproducible, precise, and incremental changes in the post TIPS portosystemic gradient. Patients such as the one we have reported, who require a TIPS revision, are critically sick and precariously poised between survival and death. Accordingly, percutaneous piecemeal placement of carefully sized covered stents deployed in an “onion skin” fashion within the hepatic venous limb of the TIPS was performed, as described above, to precisely calibrate the desired portosystemic gradient. Our technique is easily adopted, quickly employed, and offers a safe and precise alteration of the portosystemic gradient. Although this method was used for endovascular TIPS reduction purposes, the same flow reduction principles would be expected to apply elsewhere in the vascular system where flow reduction is warranted.

## Conclusions

Precision onion skin technique (POST) is a novel approach to TIPS revision, utilizing specifically sized covered stents deployed in an “onion skin” fashion. By using this method, a customized, precise hourglass narrowing within the hepatic venous limb of the TIPS creates a reproducible, desired portosystemic gradient.

## References

[1] Somberg KA, Riegler JL, LaBerge JM, Doherty-Simor MM, Bachetti P, Roberts JP, Lake JR (1995). Hepatic encephalopathy after transjugular intrahepatic portosystemic shunts: incidence and risk factors. Am J Gastroenterol.

[2] Bosch J, Abraldes JG, Berzigotti A, García-Pagan JC (2009). The clinical use of HVPG measurements in chronic liver disease. Nat Rev Gastroenterol Hepatol.

[3] Madoff DC, Wallace MJ, Ahrar K, Saxon RR (2004). TIPS-related hepatic encephalopathy: management options with novel endovascular techniques. Radiographics.

[4] Saket RR, Sze DY, Razavi MK, Kee ST, Frisoli JK, Semba CP, Dake MD (2016). TIPS reduction with use of stents or stent-grafts. J Vasc Interv Radiol.

[5] Paz-Fumagalli R, Crain MR, Mewissen MW, Varma RR (2016). Fatal hemodynamic consequences of therapeutic closure of a transjugular intrahepatic portosystemic shunt. J Vasc Interv Radiol.

[6] Haskal ZJ, Middlebrook MR (2016). Creation of a stenotic stent to reduce flow through a transjugular intrahepatic portosystemic shunt. J Vasc Interv Radiol.

[7] Madoff DC, Wallace MJ (2005). Reduced stents and stent-grafts for the management of hepatic encephalopathy after transjugular intrahepatic portosystemic shunt creation. Semin Intervent Radiol.

